# Crisis Prevention and Management during SARS Outbreak, Singapore

**DOI:** 10.3201/eid1002.030418

**Published:** 2004-02

**Authors:** Stella R. Quah, Lee Hin-Peng

**Affiliations:** * National University of Singapore

**Keywords:** severe acute respiratory syndrome, SARS, disease prevention, infectious diseases, crisis management, Asian populations, public health, perspective

## Abstract

We discuss crisis prevention and management during the first 3 months of the severe acute respiratory syndrome *(*SARS) outbreak in Singapore. Four public health issues were considered: prevention measures, self-health evaluation, SARS knowledge, and appraisal of crisis management. We conducted telephone interviews with a representative sample of 1,201 adults, ≥21 years of age. We found that sex, age, and attitude (anxiety and perception of open communication with authorities) were associated with practicing preventive measures. Analysis of Singapore’s outbreak improves our understanding of the social dimensions of infectious disease outbreaks.

An outbreak of severe acute respiratory syndrome (SARS) began in Guangdong, China, on November 16, 2002. The first three SARS cases in Singapore were confirmed on March 6, 2003. By May 5, a total of 204 cases, including 27 deaths, had been confirmed. The last case was isolated on May 11, and by July 30, the end of the outbreak, 205 patients had recovered and 33 had died (*1*).

Since SARS infection may come from ordinary contact with acquaintances, colleagues, or strangers, outbreaks can trigger anxiety and influence public perception of susceptibility, causing serious economic and social disruption. The need for health information and for crisis management by public health authorities is also high. We examine four areas of public reaction to the SARS outbreak in Singapore: preventive practices, perception of self-health, knowledge of SARS, and appraisal of SARS crisis management.

## Materials and Methods

### Sample

We interviewed a representative stratified random sample of 1,202 adults (≥21 years of age). To minimize personal contact during the outbreak, participants were interviewed by telephone instead of face-to-face. The residential telephone sampling covered 90% of households in Singapore. The response rate was 62.3%, and the sampling error ±3.5% (Table 1). We used Random Digit Dialing+1, a system commonly used in public health studies, to capture unlisted telephone numbers (*3*).

**Table 1:** Demographic characteristics of study and total population

Characteristics	Study population	Total population^a^
	No. (%) [1,201 total]	No. (%) [3,263,200 total]	
Ethnicity Chinese Malay Indian Other	900 (75.0) 172 (14.0) 82 (7.0) 47 (4.0)	2,505,400 (76.8) 453,600 (13.9) 257,800 (7.9) 46,400 (1.4)	
Age 21–29^b^ 30–39 40–49 50 and older	233 (19.0) 313 (26.0) 312 (26.0) 343 (28.0)	480,191 (20.4) 613,944 (26.1) 575,674 (24.5) 681,282 (29.0)	
Sex Male Female	599 (49.9) 602 (50.1)	1,630,293 (49.9) 1,632,916 (50.1)	

^a^Ref. 2. ^b^Total population figures refer to ages 20–29 years.

### Data Collection

We modified and expanded a structured questionnaire provided by researchers from the Department of Community Medicine, University of Hong Kong (A.J. Hedley, T.H. Tan, G.M. Leung, B.H.Y. Chan, S.Y. Ho, L.M. Ho, unpub. data). The modified questionnaire (Appendix) was translated into Mandarin, Malay, and English; interviews were conducted from May 5 to May 10, 2003. Factor analysis and logistic regression (SPSS for Windows [Version 11.5]) examined trends among four factors (SARS prevention, perception of self-health, knowledge of SARS, and perception of health authorities’ crisis management). We also assessed how prevention measures correlated with other factors, including respondents’ demographic characteristics.

### Preventive Measures

Eight questions focused on respondents’ prevention practices in the 3 days before the interview. We constructed a composite index indicating the total number (from 0 to 8) of preventive measures taken. A dichotomous indicator of preventive behavior was calculated based on the mean number of precautions taken (4.68): “low” (≤5) versus “high” (≥6).

### Self-Health Perception

Three sets of questions addressed respondents’ perception of their own health. The first set covered nine physical health complaints. We created a composite index of symptoms by adding all instances of health complaints over the previous 2 weeks. This index was 0 to 7 in our study since no one reported having more than seven of the nine symptoms.

The second set was a “frame of mind” index fashioned after B.A. Thyer’s Clinical Anxiety Scale (*4*). Scores for positive items were 1 (not at all) to 4 (very much); negative item scores were reversed, so lower total scores indicated higher anxiety. The scale had an Alpha reliability coefficient of 0.8244.

The third set addressed respondents’ perceived susceptibility to SARS. Scores were 4 (very likely) to 0 (don’t know). On the basis of the average score (1.5; standard deviation [SD] 1.01), we created a dichotomous variable to contrast respondents who believed they were susceptible to contracting SARS (scores 3 and 4) with those who did not (scores 0–2).

### Knowledge of SARS

Three questions tested SARS knowledge. Responses were scored 0 (incorrect) or 1 (correct); a composite index indicated the number of correct answers, from none correct (0) to all three correct (*3*).

### Appraisal of Crisis Management

Four sets of questions addressed respondents’ appraisal of crisis management, but we discuss the three most relevant. The first set of five questions (Alpha reliability 0.8136) assessed opinions on information distribution. Scores were 1 (very negative) to 6 (very positive). On the basis of the mean score (4.83; SD 0.617), we calculated a dichotomous index: negative appraisal (scores ≤4.7) versus positive appraisal (scores ≥4.8).

The second set of questions addressed openness of communication. Scores were 1 (very negative) to 6 (very positive). By using the sample’s mean score (4.31; SD 1.25), this variable was dichotomized into “disagreement” (scores 1–3) and “agreement” (scores 4–6).

The third set referred to the public’s acceptance of quarantine regulations. The scores were dichotomized into “agreement” (*1*) versus “no agreement” and “don’t know” (*2*).

## Results

Responses to the survey questions are summarized in Table 2. Variables were examined by using odds ratios (ORs) at 95% confidence intervals (CI). The statistically significant ORs are reported in Table 3 with their respective level of significance from the Mantel-Haenszel common odds ratio estimates.

**Table 2:** Variables used in analysis of public reaction and perspective of SARS crisis

Variable	%	Mean	SD
Symptoms (0–8) over past 2 weeks None One or more Main SARS-related symptoms Persistent high fever ≥38°C Cough Rapid breathing	77.6 22.4 1.0 9.0 1.0	0.3639	0.8286
Anxiety level High (1.0–2.2) Moderate (2.3–3.2) Low (3.3–4.0)	2.9 42.4 54.7	3.2307	.4819
Perceived likelihood of contracting SARS Very likely (4) Likely (3) Not very likely (2) Not likely at all (1) Don’t know (0)	4.0 10.0 39.0 29.0 18.0	1.5304	1.014
Knowledge of SARS No knowledge (0 of 3 answers correct) (0) 1 of 3 answers correct (1) 2 of 3 answers correct (2) 3 of 3 answers correct (3)	11.7 25.0 42.5 20.7	1.7227	.9222
Appraisal of crisis management “Strongly agree” and “Agree” that information by health authorities is: Accurate Clear Sufficient Timely Trustworthy Population has chance to express personal views and concerns to the authorities, “strongly agree” or “agree.” Agreeable to 10-day quarantine after nonclose contact with SARS-infected person and no symptoms Agree Don’t agree Don’t know	82.2 86.3 84.5 84.4 87.8 66.3 71.6 22.4 6.0		
Years of formal education ≤10 years ≥11 years	57.1 42.9	10.07	3.9642
Practice of preventive measures Practicing each of eight measures “always” or “most of the time” during the past 3 days: Covered mouth with tissue when sneezing or coughing Covered mouth with bare hand when sneezing or coughing Washed hands after sneezing, coughing, or clearing nose Used soap or liquid hand-wash when washing hands Wore a mask Used serving utensils for shared food Took preventive measures when touching objects Washed hands after touching objects Preventive measures taken over past 3 days (score 0–8) Five or fewer of the eight measures Six or more of the eight measures	62.0 47.0 72.0 81.0 4.0 28.0 15.0 48.0 69.3 30.7	4.686	1.5286

**Table 3:** Practice of SARS preventive measures, 3 days before interviews^a^

Variable	No.	OR	95% CI	
**Personal health evaluation** Symptoms in past 2 weeks None One or more Anxiety^b^ Moderate or high (score ≤3.25) Low anxiety (score >3.25) Perceived likelihood of SARS Not likely Likely	932 269 544 657 1,034 167	1.012 0.960 0.861 1.140 1.031 0.833	0.947 to 1.082 0.766 to 1.203 0.757 to 0.978 1.031 to 1.283 0.979 to 1.085 0.621 to 1.118	
**Knowledge of SARS** Two or fewer correct answers Three correct answers	952 249	1.012 0.954	0.950 to 1.079 0.753 to 1.079	
**Appraisal of crisis management** Quality of official information Below average (negative) Above average (positive) Have chance to express opinion^c^ Disagree Agree Agreeable to quarantine when non-close contact with SARS-infected person and no symptoms Agree Do not agree or don’t know	290 911 271 930 860 341	1.164 0.955 1.434 0.909 0.969 1.084	0.928 to 1.460 0.893 to 1.020 1.115 to 1.846 0.855 to 0.966 0.899 to 1.045 0.888 to 1.323	
**Demographic characteristics** Years of formal education^d^ ≤10 >10 Sex^c^ Male Female Age^c^ (y) <35 ≥35	686 515 599 602 391 809	0.909 1.143 1.339 0.770 1.365 .872	0.821 to 1.006 0.985 to 1.325 1.166 to 1.539 0.689 to 0.861 1.123 to 1.658 0.806 to 0.943	

^a^SARS, severe acute respiratory syndrome; OR, odds ratio; CI, 95% confidence interval. ^b^Asymptotic significance (2-sided) ≤0.05. ^c^Asymptotic significance (2-sided) ≤0.001. ^d^Asymptotic significance (2-sided) ≤0.10.

Recommended preventive measures were not practiced uniformly. The most practiced measures 3 days before the interview were using soap when washing hands (81%) and washing hands after sneezing, coughing, or clearing the nose (72%). The least practiced measure was wearing a mask over the mouth. A total of 4% wore masks, and most did so only when visiting a clinic or hospital or when the mask was part of a uniform (as in healthcare workers). The index of preventive measures indicates that most people (69.3%) took some preventive measures.

Respondents’ perception of their health was generally positive. A relatively low proportion (22.4%) of respondents reported having any of our nine physical health complaints over the previous 2 weeks, and fewer than 1% reported the three classic symptoms of SARS (fever ≥38°C, cough, rapid breathing). The mean number of health complaints reported in our sample was 0.369 (SD 0.828). The survey also showed low anxiety; only 2.9% of respondents reported high anxiety. The mean anxiety score was 3.23 (SD 0.48). Most respondents (68%) thought they were not very likely or not likely at all to contract SARS, and 18% were not sure of their likelihood. Those who thought they were likely to get the disease reported slightly more anxiety. Of the three aspects of health perception, only anxiety was associated with taking precautions (OR 0.861; 95% CI 0.757–0.978). In the high-anxiety group, 34% followed six or more of the eight preventive measures, in contrast to 28% of respondents who had low anxiety.

Regarding knowledge of SARS, the sample correctly answered an average of 1.722 (SD 0.922) of 3 questions on SARS transmission. Approximately 63% answered two or more questions correctly; 11.7% did not answer any questions correctly.

Respondents had a generally high opinion of authorities’ crisis management. More than 80% thought official information was accurate, clear, sufficient, timely, and trustworthy, and 72% were prepared to accept a 10-day quarantine, even in the absence of SARS symptoms or close contact with a SARS patient. Of the three crisis management aspects, one had significant influence on preventive action: respondents’ opinion of authorities’ openness to communication. People who thought that authorities were open to communication were more inclined to practice six or more of the eight SARS preventive measures (OR 0.909; 95% CI 0.855 to 0.966) than those who thought they had no chance to express their concerns to the authorities (OR 1.434; 95% CI 1.115 to 1.846).

Three demographic characteristics were associated with taking preventive measures against SARS: sex, age, and estimated years of formal education. Women were more inclined (OR 0.770; 95% CI 0.689 to 0.861) than men (OR 1.339; 95% CI 1.166 to 1.539) to take preventive measures; this finding is consistent with other studies on health behavior in Singapore (5*,**6*). People ≥35 years of age were more inclined to take preventive measures (OR 0.872; 95% CI 0.806 to 0.943) than their younger counterparts (OR 1.365; 95% CI 1.123 to 1.658). The association with education disappeared when controlled for sex.

## Discussion

Information regarding the SARS outbreak was widely distributed by the media and government; while this information was essential to keep the public informed of the risks for infection and preventive measures, it also could increase anxiety. However, we found low levels of anxiety in Singapore, and few reported health complaints. Reporting health complaints was not associated with taking precautions against SARS, possibly because the nine symptoms of SARS covered in our questionnaire are associated with other common diseases in Singapore (e.g., dengue fever, the incidence of which was 86.2 per 100,000 in May 2003) and are not usually deemed serious. In fact, familiarity with symptoms was a key initial obstacle in preventing SARS spread in hospitals (*7*) and remains an impediment to raising community alertness.

In our sample, anxiety appeared to motivate preventive behavior; those in the highest anxiety group took more precautions. However, anxiety was not associated with the perceived likelihood of contracting SARS. The low percentage of respondents who viewed SARS as a personal risk (14%, compared to 22% found in a similar survey in Toronto [*8*]) could be explained by the fact that healthcare workers were among the first SARS patients. By the time the interviews began, two physicians had died, and two hospitals had clusters of cases. Lay respondents (those with no contact with hospitals or healthcare workers) may have perceived SARS an occupational hazard.

Distribution of SARS information and prevention advice in Singapore increased rapidly over the 2 months preceding the interviews. All types of media were used, including a public television channel, the “SARS Channel,” established to give current and comprehensive information on world infection trends and Singapore’s situation. The Ministry of Health provided SARS information on its Web site (*9*), taking advantage of the fact that, as of December 2001, Singapore had 1.9 million Internet subscribers (out of 3.3 million population) (*10*). Of respondents, 20.7% were able to correctly answer all three SARS questions, and these did not differ in the practice of preventive measures from those who had less SARS knowledge. The absence of a correlation between knowledge and behavior confirms that knowledge of a disease is not sufficient to trigger preventive action (*5*,*6*,*11*–*13*).

Since SARS appeared unexpectedly, healthcare experts were uncertain how to control the epidemic. Consequently, assessing public opinion of authorities’ crisis management in our survey was relevant to Singapore. Of the aspects we examined, only public opinion of authorities’ openness to communication was correlated with taking preventive measures. The other two aspects (information dissemination and acceptance of quarantine regulations) did not affect preventive action, probably because of their very positive rating.

The public’s highly positive assessment of Singapore authorities’ crisis management is distinctive. History shows that epidemics are politically perilous to governments as, among other things, they challenge their resolve, efficiency, and state of readiness (*14*). Political leaders of other SARS-affected Asian countries witnessed this principle directly. The SARS outbreak in Singapore appears to have worked in an opposite way: it corroborated the usefulness of public health and environmental regulations. In addition, this study’s findings parallel the population’s response to quarantine and other restrictive measures, confirming previous observations of a relatively high level of social discipline in the population (*15*,*16*).

## Conclusion

Singapore was taken out of the official list of SARS-infected countries by the World Health Organization on May 30, 2003. The epidemic has left the crisis phase and entered a new phase, normalization and vigilance. As a new disease, SARS demands continuous scrutiny on all fronts, from the laboratory to the homes of the people.

## Supplementary Material

Appendix Questionnaire given to SARS survey respondents
