# Supplementary material for: Crisis Prevention and Management during SARS Outbreak, Singapore
Source: Emerg Infect Dis. 2004 Feb;10(2):364–8. doi: 10.3201/eid1002.030418 (PMC3322925; doi:10.3201/eid1002.030418)
Supplement: Appendix — Questionnaire given to SARS survey respondents [file 03-0418-app-s1.pdf]

Appendix (Online Only)

Questionnaire given to SARS survey respondents

Preventive measures

1. “Did you cover your mouth with a paper tissue or handkerchief when sneezing or coughing?”

2. “Did you cover your mouth with your bare hand when sneezing or coughing?”

3. “Did you wash your hands after sneezing, coughing, or cleaning your nose in the past three days?”

4. “Did you use soap or liquid hand-wash when washing your hands in the past three days?”

5. “Did you wear a mask over your mouth in the past three days?”

6. “Did you use serving utensils (chopsticks or spoons) for shared food when joining others over the past three days?”

7. “In the past three days, when touching objects that might possibly carry the SARS virus [e.g., door handles, buttons in the lifts], did you take preventive measures (e.g., pressing lift buttons with tissue paper)?”

8. “In the past three days, after touching objects that might possibly carry the SARS virus [e.g., door handles, buttons in the lifts], did you wash your hands as soon as possible?”

Responses

(a) Always

(b) Most of the time

(c) Sometimes

(d) Not at all

Personal health evaluation

Physical health complaints

“In the past 2 weeks, have you had any of the following symptoms?”

1. Persistent high fever of 38°C (100.4°F) or higher, lasting for a day or more

2. Feeling cold

3. Headache

4. Having aches all over the body

5. Cough

6. Rapid breathing

7. Dizziness

8. Running nose

9. Sore throat

Responses

(a) Yes

(b) No

(c) Don’t know

Frame of mind

(a) Very much

1. “Thinking of the way you feel these days, would you say you feel at ease?”

2. “Thinking of the way you feel these days, would you say you feel contented?”

3. “Thinking of the way you feel these days, would you say you feel comfortable?”

4. “Thinking of the way you feel these days, would you say you feel relaxed?”

5. “Thinking of the way you feel these days, would you say you feel happy?”

6. “Thinking of the way you feel these days, would you say you feel frightened?”

7. “Thinking of the way you feel these days, would you say you feel nervous?”

8. “Thinking of the way you feel these days, would you say you feel anxious?”

9. “Thinking of the way you feel these days, would you say you feel indecisive?”

10. “Thinking of the way you feel these days, would you say you feel confused?”

(b) Quite

(c) Just a little

(d) Not at all

Perceived susceptibility to SARS

“How likely do you think it is for you to contract SARS?”

(a) Very likely

(b) Likely

(c) Not very likely

(d) Not likely at all

(e) Don’t know

Knowledge of SARS

1. “In your opinion, is SARS transmitted through saliva droplet transmission?”

2. “In your opinion, is SARS transmitted through airborne transmission?”

3. “In your opinion, is SARS transmitted through hand-contact transmission?”

Responses

(a) Yes

(b) No

(c) Don’t know

Appraisal of Crisis Management

Official Crisis Information

1. “With regards to the distribution of information by the health authorities to the public in Singapore, do you agree or disagree that it has generally been accurate?”

2. “With regards to the distribution of information by the health authorities to the public in Singapore, do you agree or disagree that it has generally been clear?”

3. “With regards to the distribution of information by the health authorities to the public in Singapore, do you agree or disagree that it has generally been sufficient?”

4. “With regards to the distribution of information by the health authorities to the public in Singapore, do you agree or disagree that it has generally been timely?”

Responses

(a) Strongly disagree

(b) Disagree

(c) Not sure, but probably disagree

(d) Not sure, but probably agree

(e) Agree

(f) Strongly agree

5. “With regards to the distribution of information by the health authorities to the public in Singapore, do you agree or disagree that it has generally been trustworthy?”

Openness of Communication

“Do you agree or disagree that you have had the chance to express your personal views and concerns to the authorities if you wanted to?”

(a) Strongly disagree

(b) Disagree

(c) Not sure, but probably disagree

(d) Not sure, but probably agree

(e) Agree

(f) Strongly agree

Acceptance of Regulations

“If you did not develop symptoms of SARS after having nonclose contact with someone diagnosed with SARS, would you agree to be quarantined for 10 days?”

(a) Agree

(b) Don’t agree

(c) Don’t know
